# Calling Phenology of Two Frog Species in South Korean Rice Paddies Using Automated Call Detection

**DOI:** 10.3390/ani15213141

**Published:** 2025-10-29

**Authors:** Soyeon Chae, Jinu Eo, Yikweon Jang

**Affiliations:** 1 Division of EcoScience, Ewha Womans University, Ewhayeodaegil-52, Seodaemun-gu, Seoul 03760, Republic of Korea; 2 National Institute of Agricultural Sciences, Rural Development Administration, Wanju 55365, Republic of Korea; 3 Department of Life Sciences, Division of Ecoscience, Ewha Womans University, Ewhayeodaegil-52, Seodaemun-gu, Seoul 03760, Republic of Korea

**Keywords:** frog calling phenology, passive acoustic monitoring, automated call detection, *Dryophytes japonicus*, *Pelophylax nigromaculatus*, rice paddies, weather effects

## Abstract

**Simple Summary:**

Amphibians are sensitive to changes in their surroundings, and the phenology of breeding calls can reflect how they respond to weather and farming practices. We studied two common frog species in South Korean rice paddies over five years using sound recorders. This made it possible to track calling activity over long periods without disturbing the animals. *Dryophytes japonicus* showed a short and synchronized calling peak in early summer, while *Pelophylax nigromaculatus* began earlier in spring and continued calling for longer, although at lower levels. Temperature was the main factor driving calling in both species, with the *D. japonicus* showing especially strong responses. Humidity also played a role, particularly for the *P. nigromaculatus*, which called more actively when conditions were moist. These results reveal how frogs adapt their reproductive behavior to agricultural wetland environments and changing weather conditions. Our study highlights the ecological value of rice paddies as breeding habitats and shows that automated acoustic monitoring can be a powerful tool for long-term biodiversity observation and conservation planning.

**Abstract:**

Amphibian breeding phenology provides key insights into species’ sensitivity to climatic and anthropogenic drivers. We used passive acoustic monitoring (PAM) with automated call detection to examine the calling activity of *Dryophytes japonicus* and *Pelophylax nigromaculatus* in South Korean rice paddies across five breeding seasons (2018–2022). Both species exhibited distinct seasonal patterns: *D. japonicus* showed a synchronous and concentrated calling peak in mid-June (GAM deviance explained = 34%), whereas *P. nigromaculatus* initiated calling earlier and maintained a longer, less synchronized calling period extending into July (GAM deviance explained = 19%). Zero-inflated negative binomial models demonstrated that temperature was the strongest predictor of calling activity in both species, though responses to humidity and wind differed. *D. japonicus* maintained high calling rate under warm conditions, with only modest suppression at high humidity, whereas *P. nigromaculatus* was strongly inhibited by combined warm and humid conditions. These results establish a detailed information on the calling phenology of *D. japonicus* and *P. nigromaculatus* in East Asian agroecosystems highlight species-specific sensitivities to local weather variables. Our findings demonstrate that automated acoustic monitoring offers an efficient way to document ecological responses to weather variability and may serve as a long-term tool to track phenological shifts under climate change. Future advances in sound analysis, including the integration of deep-learning algorithms and cross-species detection frameworks, could further improve automated biodiversity monitoring in complex agricultural landscapes.

## 1. Introduction

Amphibian breeding phenology is the seasonal timing of reproductive activity, and it is a critical biological process that reflects environmental sensitivity. Because amphibians rely on external environmental cues to initiate breeding, shifts in temperature, precipitation, humidity, and hydrological conditions directly influence calling activity and spawning [1,2]. Unlike many vertebrates that can buffer environmental changes behaviorally or physiologically, amphibians’ permeable skin and dependence on aquatic habitats make them acutely responsive to short-term climatic fluctuations [3]. As such, amphibians are considered bioindicators of climate change, and their phenological shifts provide early warnings of broader ecosystem responses [4,5].

Globally, evidence has accumulated that amphibian breeding phenology is shifting under ongoing climate change. Increasing spring temperatures often lead to earlier onset of calling and breeding in temperate frog species [6,7]. For example, long-term datasets from North America and Europe reveal that hylid and ranid frogs now initiate choruses several weeks earlier than in past decades [8,9]. Yet these responses are not universal: some populations exhibit delayed or inconsistent breeding linked to altered precipitation regimes or reduced snowpack, while others display non-linear patterns where initial advances plateau over time [10,11]. Similarly, in East Asia, monitoring of *Dryophyte japonicus* and *Dryophyte suweonensis* in rice paddies has revealed cases where reproductive activity appeared later than expected under rising spring temperatures, likely reflecting the overriding influence of precipitation cues and agricultural water management [12]. These variable responses highlight the complexity of phenological processes, where temperature interacts with moisture availability, habitat type, and local climatic variability. Such global patterns raise important questions about how amphibians respond in intensively managed agroecosystems, particularly in East Asia.

Key environmental drivers operate at multiple temporal scales. Temperature accelerates gonadal development and metabolic rates, directly influencing the readiness of males to initiate calling [1]. Rainfall events create or replenish aquatic habitats, acting as immediate triggers for explosive choruses in many species [13]. High relative humidity reduces desiccation stress during prolonged calling bouts, while wind speed interferes with sound transmission, increasing energetic costs and decreasing effective mate attraction [14,15]. These drivers not only shape daily calling activity but also scale up to determine seasonal onset, peak, and termination of reproductive periods. Importantly, such relationships suggest that amphibian phenology is an integrative outcome of multiple, potentially interacting climatic factors rather than a simple function of temperature alone [3,8]. In East Asia, these climatic drivers intersect strongly with agricultural environments, where amphibians often depend on human-modified wetlands.

In East Asia, rice paddies serve as surrogate wetlands and have become critical breeding habitats for amphibians following widespread loss of natural wetlands [16]. These artificial systems are doubly influenced: they are exposed to climatic variability while also subject to intensive human management through irrigation schedules, water-level control, pesticide use, and mechanized farming [17,18]. These management practices not only alter habitat availability but also reshape the acoustic environment, adding layers of anthropogenic and geophonic interference to amphibian communication. As a result, amphibian calling activity in paddies may deviate from patterns observed in natural wetlands, reflecting the combined influences of climate and agricultural practices [19,20,21,22]. Pesticide exposure can disrupt endocrine and vocal systems relevant to calling and reproduction [23,24,25] and anthropogenic noise interferes with acoustic communication [26,27,28].

*Dryophytes japonicus* (Japanese treefrog) and *Pelophylax nigromaculatus* (Black-spotted Pond frog) are two of the most widespread amphibian species inhabiting East Asian rice paddies. *D. japonicus* is a small arboreal hylid that breeds in shallow, vegetated paddy margins and produces short, high-pitched tonal calls concentrated in early summer [16,29]. In contrast, *P. nigromaculatus* is a larger ranid species that breeds in open paddy fields and emits low-frequency, pulsed calls with strong harmonic energy [13,30]. These ecological and acoustic contrasts make the two species suitable model taxa for examining how climatic and agricultural factors shape calling phenology in agroecosystems. Both species are abundant in agricultural wetlands across East Asia and rely on advertisement calls for reproduction. *D. japonicus* produces tonal advertisement calls with a fundamental frequency of approximately 1.7 kHz, each note lasting 0.10–0.20 s and separated by intervals of 0.20–0.50 s [31,32]. In contrast, *P. nigromaculatus* emits advertisement calls characterized by a dominant frequency of about 1.9 kHz (mean 1932 ± 367 Hz), with each call lasting around 0.39 ± 0.07 s. Calls typically consist of 3–5 pulse groups per call, and each group contains approximately 4–10 pulses [30].

Passive Acoustic Monitoring (PAM) has become a transformative approach in ecological research, providing a non-invasive, scalable, and cost-effective method for biodiversity monitoring across extended temporal and spatial scales [33,34]. PAM allows for continuous acoustic data collection, which is especially valuable for species that are nocturnal, elusive, or sensitive to human presence. Amphibians are among the most relevant groups for acoustic monitoring because many species rely heavily on species-specific advertisement calls during the breeding season. These vocalizations not only reveal species presence but also serve as proxies for abundance and phenological patterns, offering insights into reproductive timing and seasonal dynamics [35,36].

The major challenge of PAM is not data collection but data processing. A single recording unit deployed for several months can generate thousands of hours of audio, making manual annotation impractical for long-term monitoring projects [33]. To address this, researchers have developed automated approaches, including acoustic indices, clustering algorithms, and machine learning classifiers, to efficiently extract ecological information from large datasets [37,38]. These tools reduce massive raw audio into manageable forms that can inform ecological understanding. However, automated detection often faces difficulties in complex environments, where geophony (wind, rain, water movement), biophony (insects, birds, non-target amphibians), and anthropophony (e.g., agricultural machinery) overlap and mask target calls, lowering detection accuracy [35,37].

These challenges are particularly pronounced in agricultural landscapes such as rice paddies, which dominate much of East Asia’s farmland. Rice paddies serve as surrogate wetlands for amphibians after widespread natural wetland loss [16], but open landscapes increase wind noise, fluctuating water levels add variable geophonic signals, and farming activities introduce strong anthropogenic interference [17,27,35]. Automated detectors that work well in forests or undisturbed habitats often show reduced accuracy in these agricultural soundscapes. Nevertheless, PAM provides high-resolution temporal datasets that are difficult to obtain with manual surveys, enabling analyses of amphibian phenology at daily, seasonal, and interannual scales. Despite its potential, few studies have established long-term phenological baselines for amphibians in East Asian rice paddies, leaving a critical gap in understanding how climate and agriculture jointly shape reproductive timing [18,39].

The present study integrates automated acoustic monitoring with ecological analysis to address three primary objectives: (1) to describe the seasonal and interannual phenological patterns of *D. japonicus* and *P. nigromaculatus* across multiple years in Korean rice paddies, (2) to examine site-level variation in phenological pattern, and (3) to evaluate how environmental factors, particularly temperature, humidity, and wind, influence their calling dynamics. We hypothesize that calling activity correlates with latitude, such that lower-latitude sites are expected to initiate calling earlier than higher-latitude sites. In this study, the onset of calling refers to the first detection date of vocal activity, which does not necessarily indicate the onset of breeding. By combining species-specific detection with statistical modeling, this research establishes a detailed ecological baseline for reproduction of two frog species: *D. japonicus* and *P. nigromaculatus*. Ultimately, these results contribute to a broader understanding of how reproductive phenology in amphibians is shaped by the interaction between climate variability and agricultural landscapes.

## 2. Materials and Methods

### 2.1. Recording and Target Species

**Figure 1:**
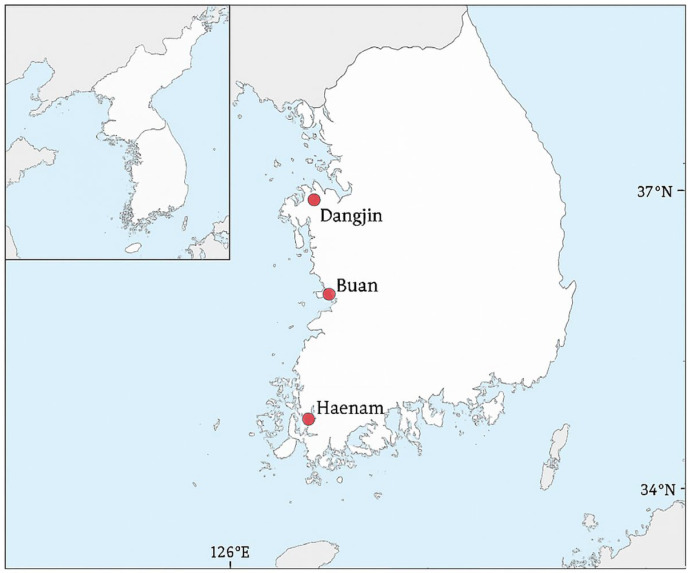
Study sites of passive acoustic monitoring in South Korea. The three sites (Dangjin, Buan, and Haenam) in red were all rice paddies. The sites are distributed along the west and south coasts, spanning approximately one degree of latitude. Base map data: Natural Earth (public domain).

Passive acoustic monitoring was conducted in rice paddy wetlands at three study sites in the Republic of Korea: Dangjin, Buan and Haenam (Figure 1). Monitoring was conducted during the breeding seasons from March to August in each year between 2018 and 2022. These paddy sites were created through coastal reclamation and are managed through irrigation and drainage systems that maintain shallow standing water throughout the breeding season. They are embedded in intensive agricultural landscapes common across lowland Korea, with vegetated margins and surrounding farmland that provide suitable conditions for amphibian breeding. Importantly, the three study sites are distributed along the west and south coasts of Korea and span approximately one degree of latitude, providing a natural gradient in climatic conditions that can influence amphibian breeding activity.

At each study site, a single rice paddy was selected for monitoring and one autonomous recorder (Song Meter SM4; Wildlife Acoustics Inc., Maynard, MA, USA) was installed near the center of the paddy at a height of approximately 1.5–1.8 m above the ground. Microphones were equipped with foam windscreens to minimize wind noise. Recorders were programmed to operate twice per day, from 17:30 to 19:30 and from 21:20 to 23:30 (Korea Standard Time). The first window captures the beginning of evening calling activity that follows sunset, and the second window covers the late-evening peak when calling activity is highest and anthropogenic noise in paddies is minimal. Audio files were stored in uncompressed WAV format with a sampling rate of 44.1 kHz, 16-bit resolution, and mono channel mode. Due to unexpected recorder malfunction and power interruptions, some recording dates had missing or corrupted files, which were excluded from the analysis (Table 1).

Two frog species were analyzed in this study: *D. japonicus* and *P. nigromaculatus*. These well-documented acoustic features including frequency range, duration, and pulse-group structure, enable species-specific detection through automated analytical methods.

### 2.2. Call Detection

To establish a reference dataset for automated detection, we randomly selected approximately 5% of all recordings, ensuring balanced coverage across sites, months, and diel periods. Each selected file was manually annotated in Raven Pro 1.6 (Cornell Lab of Ornithology). Spectrograms were visually examined, and advertisement calls of *P. nigromaculatus* and *D. japonicus* were manually annotated by marking their start and end times. From this annotated subset, 10% of files were used as a training set to guide parameter optimization, and the remaining 90% formed an independent test set for performance evaluation.

Automated detection for both species was performed using Non-Negative Matrix Factorization (NMF) implemented in the soundscape_IR toolbox [40]. A range of parameter settings was tested, including FFT size (256, 512, 1024), prewhiten percentage (50–80%), window overlap (0.25, 0.5, 0.75), and analysis frequency band. The results were summarized in a confusion matrix, and accuracy, precision and recall were calculated. Among all tested parameter combinations, the configuration yielding the highest detection accuracy against manual annotations was selected (FFT = 1024, prewhiten = 50%, overlap = 0.75). This configuration provided the best trade-off between frequency resolution and detection performance and was applied to the full dataset for subsequent analyses.

### 2.3. Species Level Phenology

To characterize temporal variation in calling activity, detected calls were first summarized by year and site to construct phenological patterns for *D. japonicus* and *P. nigromaculatus*. Recording dates were converted to Julian days (1–365) to provide a continuous temporal index across years and to facilitate alignment of seasonal patterns. Seasonal and interannual trends were examined by visualizing both monthly and daily distributions of calling activity. Non-parametric statistical tests were then applied to assess phenological differences. Kruskal–Wallis tests were used to evaluate monthly differences in call counts during the peak season (May–July), followed by post hoc Mann–Whitney U tests with Bonferroni correction for pairwise comparisons. Interannual variation was further examined by comparing peak-season (June) call counts across years using the same procedure. These tests quantified both seasonal concentration and year-to-year variability in calling rate (calls per recorder-day).

To model non-linear seasonal trends in daily calling activity, we fitted generalized additive models (GAMs) using a Gaussian error distribution and smoothing splines for day of year. GAMs are well suited for phenological data because they flexibly capture curved temporal patterns that cannot be described by linear models [41]. Daily dynamics of calling activity were further explored using smoothing approaches. GAMs with Julian day as a smooth predictor were fitted separately for each species to characterize within-season calling curves. This approach allowed estimation of the timing and shape of seasonal peaks beyond discrete monthly comparisons, providing a flexible framework to detect non-linear responses. Model fit was evaluated using Akaike Information Criterion (AIC) and residual diagnostics. Statistical significance of smooth terms was assessed at α = 0.05. All analyses were conducted in RStudio (4.2.2) using the packages lme4 (1.1-37), glmmTMB (1.1.13), mgcv (1.9-3), and stats (4.5.1).

### 2.4. Site Level Phenology

Recording effort varied among months and sites. Therefore, all site-level analyses were based on daily call counts limited to days with recordings, expressed as calls per recording day. Calling rate was calculated as the number of species-specific detections per 24 h recorder-day. For each species, site-specific monthly time series for the breeding season (March–August, 2018–2022) were generated by aggregating daily counts within site × month × year strata; excluding days without recordings. Spatial variation in calling rate was examined descriptively using daily call counts during the peak breeding period (May–July). Median and interquartile ranges were summarized to compare relative activity among Dangjin, Buan, and Haenam. Given that only three recording sites were available, no formal statistical tests between sites were conducted; comparisons were limited to descriptive summaries and visual inspection of site-level differences. To summarize seasonal timing at each site, cumulative-activity thresholds (10%, 50%, and 90% of total seasonal calls) were calculated to represent onset, peak, and termination dates based on Julian day. These indicators were computed for each site × year and reported as mean ± SD across years (2018–2022). Because of the limited number of spatial replicates, these timing metrics were interpreted descriptively to assess regional synchrony. All site-level summaries and visualizations were conducted in Python (3.10.9) using pandas, numpy, and SciPy.

### 2.5. Environmental Correlates of Calling Activity

To assess environmental drivers of calling activity, daily meteorological data were obtained from the National Institute of Agricultural Weather Service (https://weather.rda.go.kr/weather/observationInfo.do; accessed on 1 March 2025). For Buan and Haenam, we used local meteorological data from within each region, while for Dangjin, data from the nearest station in Taean, located within the same Chungcheongnam-do province, were used. Variables included minimum, maximum, and mean air temperature (°C), relative humidity (%), wind speed (m/s), and daily precipitation (mm). Generalized linear mixed models (GLMMs) were employed with daily call counts as the response variable. Poisson error structures were initially applied, and negative binomial models were fitted when overdispersion occurred. Zero-inflated models (ZIP/ZINB) were considered in cases of excess zeros. Fixed effects included temperature, humidity, wind speed, and precipitation, while site identity and recording date were included as random effects to account for repeated measures and spatial structure. Interaction terms, such as temperature × humidity, were tested to capture conditional effects. Model performance was evaluated using Akaike Information Criterion (AIC), likelihood ratio tests, and residual diagnostics, and statistical significance was assessed at α = 0.05.

## 3. Results

### 3.1. Automated Detection Performance Across Species

Applying the NMF-based detection framework to our recordings, we assessed model performance using confusion-matrix–based metrics, reporting accuracy, precision, and recall for each species (Table 2). It reached an accuracy of 74% for *P. nigromaculatus* (precision = 0.40, recall = 0.40) and 87% for *D. japonicus* (precision = 0.81, recall = 0.81). These results suggest that an NMF-based, species-tailored approach can perform reliably under field noise conditions. The relatively higher accuracy for *D. japonicus* may reflect its more distinctive spectral structure, whereas the lower performance for *P. nigromaculatus* is likely due to overlap with background noise and sympatric vocalizations.

### 3.2. Species-Specific Calling Phenology

**Figure 2:**
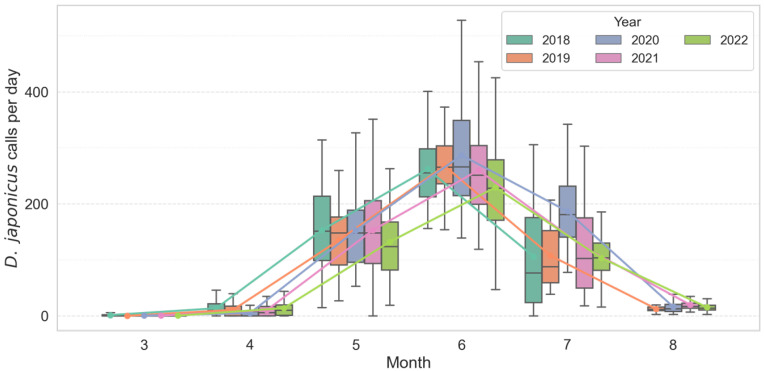
Seasonal and inter-annual variation in monthly calling activity of *D. japonicus* across multiple breeding seasons (2018–2022). Colored boxes show interquartile ranges (25th–75th percentiles), horizontal lines inside boxes denote medians, and whiskers represent 1.5 × IQR. Colored lines indicate per-year monthly mean trends, highlighting the consistent June peak and abrupt seasonal onset in *D. japonicus*.

**Figure 3:**
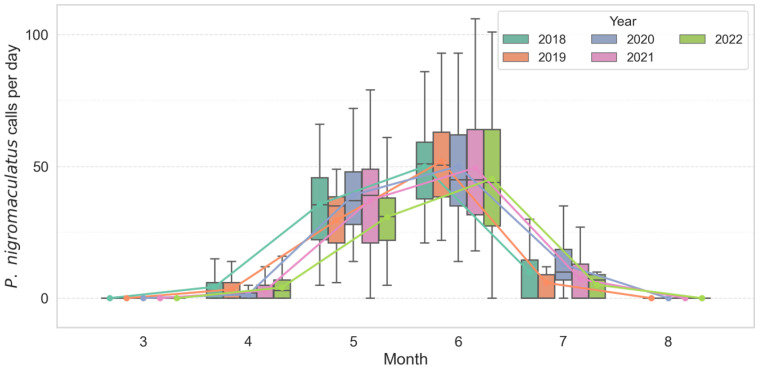
Seasonal and inter-annual variation in monthly calling activity of *P. nigromaculatus* across multiple breeding seasons (2018–2022). Colored boxes show interquartile ranges (25th–75th percentiles), horizontal lines inside boxes denote medians, and whiskers represent 1.5 × IQR. Colored lines indicate per-year monthly mean trends, illustrating the gradual early-season increase (April–May) and earlier onset pattern relative to *D. japonicus*.

Analysis of monthly call count distributions revealed strong seasonal and interannual variation in both *D. japonicus* and *P. nigromaculatus*. *D. japonicus* exhibited a sharp increase in activity from late May, with a pronounced peak consistently observed in June across all years (Figure 2). In June, the species produced a mean of 271.8 ± 68.2 calls/day (median = 262.5, range = 145–535, *n* = 450 recorder-days). Annual means ranged from 229.2 ± 85.8 in 2022 to 290.2 ± 88.7 in 2020, but median values exceeded 200 calls/day in every year, confirming synchronous breeding choruses. Statistical comparisons confirmed that June activity was significantly higher than in May and July (Kruskal–Wallis χ^2^ = 510.71, df = 2, *p* < 0.001; post hoc Mann–Whitney with Bonferroni correction, *p* < 0.01). In contrast, *P. nigromaculatus* showed an earlier seasonal onset, with calling activity emerging in April–May, reaching its highest levels in June, and tapering off by mid-July (Figure 3). In June, this species produced a mean of 52.7 ± 17.3 calls/day (median = 51.0, range = 21–136, *n* = 450). Annual means varied from 45.3 ± 25.3 in 2022 to 56.4 ± 20.7 in 2019. Seasonal differences were significant (Kruskal–Wallis χ^2^ = 817.76, df = 2, *p* < 0.001), with all pairwise comparisons between months also significant (*p* < 0.001).

**Figure 4:**
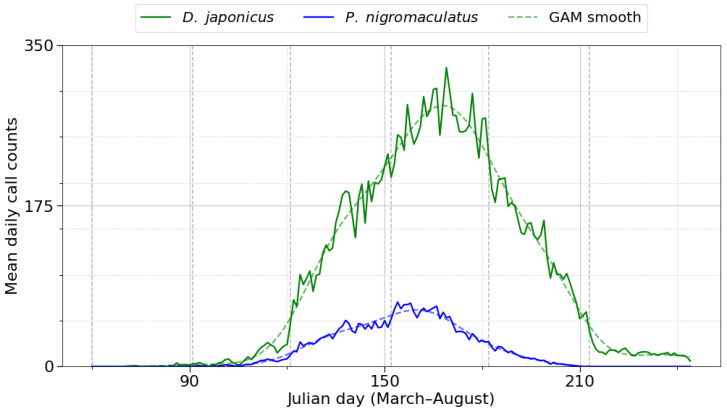
Daily calling phenology of *D. japonicus* (green) and *P. nigromaculatus* (blue) across breeding seasons (2018–2022). The solid lines represent raw daily call counts, dashed lines represent generalized additive model (GAM) smooths, confirming distinct within season dynamics. Vertical dashed lines mark the onset of each month (March–August). *D. japonicus* exhibited a sharp peak in early June, whereas *P. nigromaculatus* showed earlier onset and a broader, less synchronized calling period extending into July.

Generalized additive modeling (GAM) of daily call counts characterized within-season dynamics for the two focal species (Figure 4). *D. japonicus* reached its maximum on Julian day 169 (June 18), with mean daily calling activity of 325 calls. Activity increased from late May and declined after mid-July. The spread of activity in the raw data was SD = 23.8 days and IQR = 30 days. *P. nigromaculatus* reached its maximum earlier, on Julian day 154 (June 3), with a mean of 70 calls per day. Its activity started in late April, remained elevated through early to mid-June, and decreased toward mid-summer (SD = 18.7 days, IQR = 26 days). Model fits explained 34% of deviance for *D. japonicus* and 19% for *P. nigromaculatus* (both *p* < 0.001), indicating that *D. japonicus* exhibited a more synchronized and predictable seasonal peak, whereas *P. nigromaculatus* showed a broader, less sharply synchronized seasonal pattern.

### 3.3. Site-Level Variation in Calling Phenology

**Figure 5:**
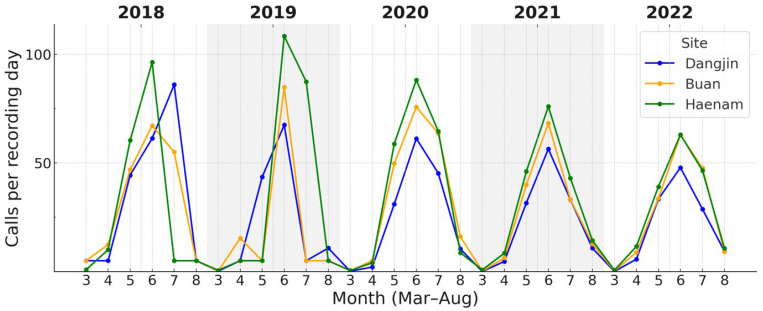
Monthly calling rates (calls per recording-day) of *D. japonicus* across three sites (Dangjin, Buan, Haenam) during 2018–2022. Colored lines show site-specific monthly medians within the breeding season of each year. The figure illustrates regional variation in calling rates, with generally higher values at Haenam, intermediate at Buan, and lower at Dangjin. Detailed seasonal timing based on cumulative-activity thresholds is provided in Table 3.

**Figure 6:**
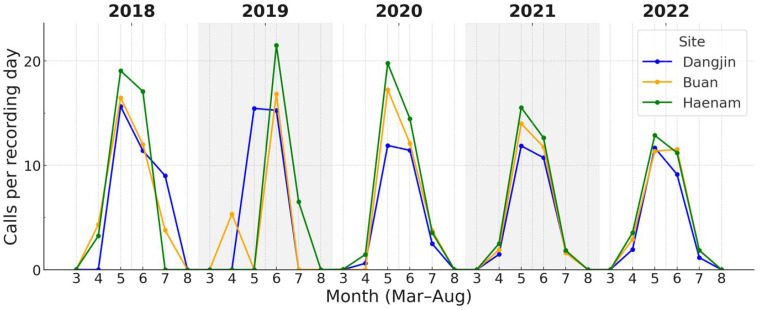
Monthly calling rates of *P. nigromaculatus* across the three sites (2018–2022). Onset occurred earlier (April–May) and peaks were broader than in *D. japonicus*. Haenam frequently (in 2019, 2021, and 2022) attained the highest rates, Dangjin the lowest, with Buan intermediate.

Across the three study sites, both focal species showed variation in the magnitude of calling activity during the breeding season. For *D. japonicus*, daily call counts during the peak period (May–July) showed clear site-related differences (Figure 5). Call rates tended to be higher at Haenam, lower at Dangjin, with Buan intermediate. For *P. nigromaculatus*, overall site differences were also evident, with the contrast being most pronounced between Dangjin and Haenam, while Buan occupied an intermediate position (Figure 6).

Cumulative-activity thresholds showed differences in phenological timing (Table 3). For *D. japonicus*, onset (10% cumulative activity) occurred earlier at Haenam (134.0 ± 2.4) and Buan (134.8 ± 2.3) than at Dangjin (136.2 ± 1.5), with peaks (50%) around day 166–168 and termination (90%) between days 197–199 across sites. Similarly, *P. nigromaculatus* began calling earlier at Haenam (129.0 ± 2.4) and Buan (129.8 ± 1.9) than at Dangjin (131.6 ± 1.9), with peaks around day 156–157 and termination near day 180 at all sites.

Overall, first detections and cumulative onset dates differed by 1–3 days among sites, but all converged on peaks in early to mid-June, indicating broadly synchronized timing across regions. In contrast, calling rate varied more noticeably, with median daily counts about twice as high at Haenam as at Dangjin.

### 3.4. Correlation Between Calling Activity and Weather Variables

**Figure 7:**
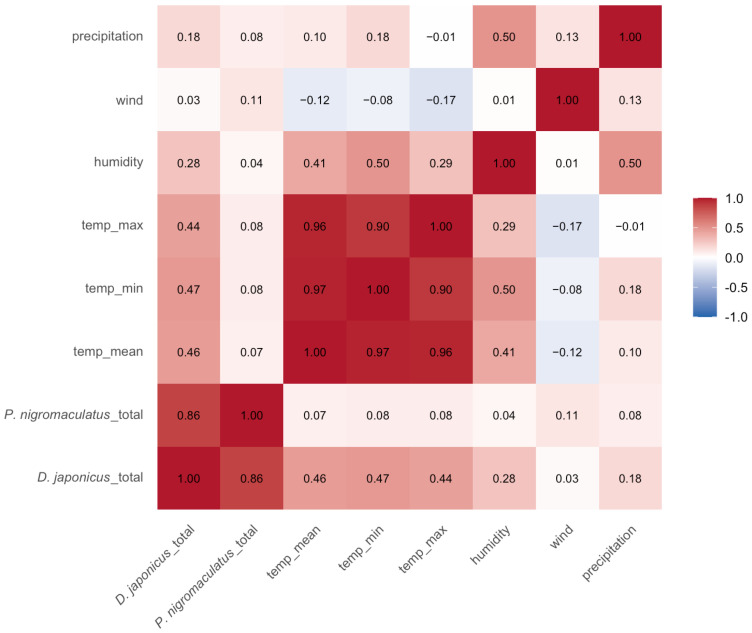
Spearman correlation matrix between daily calling activity of *D. japonicus* and *P. nigromaculatus* and local weather variables (mean, minimum, and maximum daily temperature, humidity, wind speed and precipitation). The analysis was conducted using nonzero daily call counts across all sites and years. Calling activity of *D. japonicus* was positively associated with temperature and humidity, while *P. nigromaculatus* showed weak or negligible correlations with all tested weather variables.

Spearman correlation analysis revealed distinct relationships between daily calling activity and weather conditions (Figure 7). For *D. japonicus*, call counts were moderately and positively correlated with daily mean air temperature (ρ = 0.46), minimum temperature (ρ = 0.44), maximum temperature (ρ = 0.47), and relative humidity (ρ = 0.28), whereas wind speed showed no meaningful relationship (ρ = 0.03). In contrast, *P. nigromaculatus* exhibited generally weak correlations with weather variables, with negligible associations with temperature (ρ ≤ 0.08) and humidity (ρ = 0.04). These results indicate that *D. japonicus* calling activity is strongly influenced by temperature and to a lesser extent humidity, whereas *P. nigromaculatus* appears relatively insensitive to short-term weather variation.

As expected, the weather variables themselves were highly inter-correlated, particularly among the temperature metrics (ρ = 0.90–0.97). This collinearity suggests that temperature should be represented by a single variable (e.g., mean temperature) in subsequent modeling analyses (e.g., GLMM), to avoid multicollinearity issues. Although precipitation was moderately correlated with humidity (ρ = 0.50), it was excluded from subsequent GLMM analyses because heavy rainfall events introduced background noise that interfered with acoustic call detection, potentially biasing the count data.

GLMM analysis indicated that *D. japonicus* calling activity was significantly influenced by mean temperature (β = 0.47 ± 0.002, *p* < 0.001), humidity (β = 0.17 ± 0.002, *p* < 0.001), and wind speed (β = 0.07 ± 0.002, *p* < 0.001). In addition, a strong negative interaction between temperature and humidity was detected (β = −0.33 ± 0.003, *p* < 0.001), suggesting that the positive effect of temperature on calling activity was diminished under humid conditions. While the Poisson GLMM was initially selected as the best-fitting model based on AIC, diagnostic tests revealed a high degree of zero inflation (*p* < 0.001), indicating that zero-inflated models (ZIP/ZINB) provide a more appropriate framework for capturing the data structure.

ZINB models further clarified species-specific responses. For *D. japonicus*, calling activity was strongly and positively associated with mean daily temperature (β = 1.70 ± 0.06, *p* < 0.001), whereas humidity had no significant main effect (*p* = 0.46). Wind speed exerted a modest positive effect (β = 0.16 ± 0.03, *p* < 0.001). A strong negative temperature × humidity interaction (β = −0.91 ± 0.05, *p* < 0.001) suggested that the stimulatory effect of temperature on calling activity diminished under humid conditions. In the zero-inflation component, wind speed significantly increased the probability of non-calling days (β = 1.84 ± 0.51, *p* < 0.001), whereas humidity showed no effect.

For *P. nigromaculatus*, ZINB models indicated that both mean temperature (β = 0.63 ± 0.07, *p* < 0.001) and humidity (β = 0.14 ± 0.03, *p* < 0.001) were significant positive predictors of calling activity, whereas wind speed had no effect (*p* = 0.69). A strong negative interaction between temperature and humidity (β = −1.04 ± 0.05, *p* < 0.001) revealed that calling activity was suppressed under simultaneous warm and humid conditions. In the zero-inflation component, humidity increased the probability of non-calling days (β = 0.31 ± 0.06, *p* < 0.001), whereas wind decreased it (β = −0.24 ± 0.05, *p* < 0.001).

**Figure 8:**
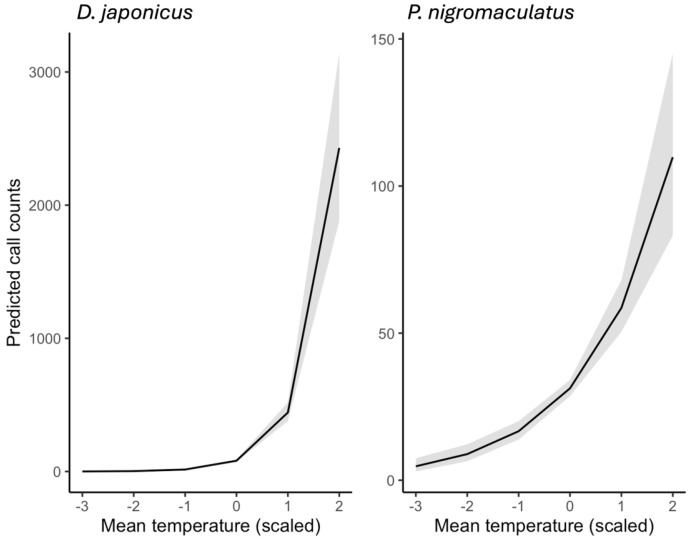
Predicted effects of mean daily temperature on the calling activity of *D. japonicus* and *P. nigromaculatus*, based on zero-inflated negative binomial (ZINB) GLMMs. Temperature values are standardized (z-scores), with 0 corresponding to the mean observed temperature across all recordings (2018–2022). Solid lines show model predictions, and shaded bands represent 95% confidence intervals.

**Figure 9:**
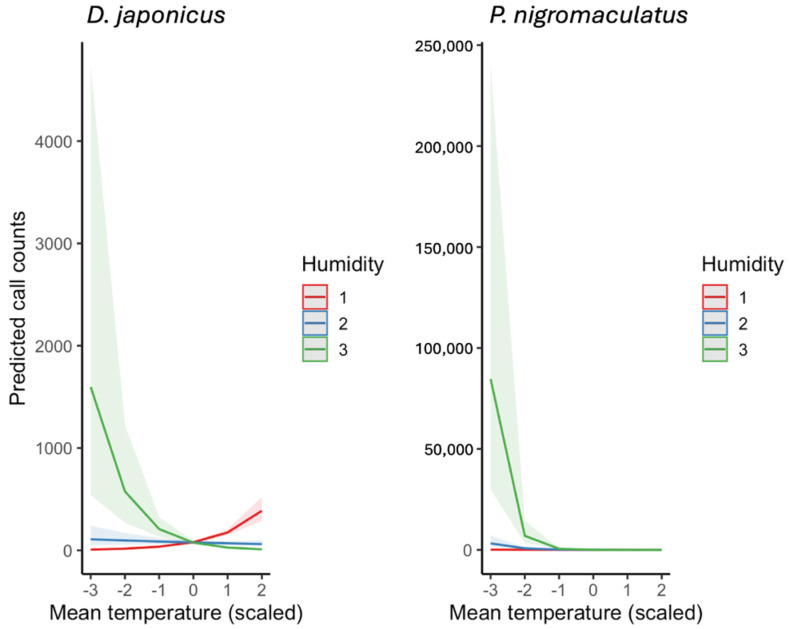
Predicted interactive effects of mean daily temperature and humidity on the calling activity of *D. japonicus* and *P. nigromaculatus*, derived from ZINB GLMMs. Humidity was divided into three levels of the observed distribution for visualization: low (−1 SD, labeled as Humidity 1), average (Humidity 2), and high (+1 SD, Humidity 3). Lines represent model predictions, and shaded bands indicate 95% confidence intervals.

Model predictions from ZINB GLMMs revealed clear effects of weather on amphibian calling activity (Figure 8 and Figure 9). When examined separately, both species showed strong positive responses to rising temperature (Figure 8). *D. japonicus* reached predicted means of 2800–3200 calls/day under the warmest observed conditions (mean temperature ≈ +2 SD), whereas *P. nigromaculatus* peaked at 90–120 calls/day under comparable conditions. These differences highlight the much higher calling rate and synchrony of *D. japonicus* compared to *P. nigromaculatus*.

Beyond main effects, a pronounced interaction between temperature and humidity was detected (Figure 9). For visualization, daily mean relative humidity was divided into three representative levels of the observed distribution: low (−1 SD, Humidity 1), average (Humidity 2), and high (+1 SD, Humidity 3). In *D. japonicus*, the stimulatory effect of temperature on calling activity was clearly reduced under high humidity, with predicted call counts plateauing even under warm conditions. In *P. nigromaculatus*, this suppression was even stronger: high humidity substantially dampened calling activity at elevated temperatures, reducing predicted counts to near baseline levels.

Overall, these models indicate that temperature is the primary driver of calling activity in both species, but humidity modifies these responses in species-specific ways. *D. japonicus* maintains high calling rate unless humidity is extreme, whereas *P. nigromaculatus* is far more sensitive to combined warm and humid conditions, which strongly suppress calling behavior.

## 4. Discussion

This study demonstrates that an NMF-based automated detection framework, when tuned appropriately, can provide reliable classification of amphibian calls in natural environments. Detection accuracy reached 74% for *P. nigromaculatus* and 87% for *D. japonicus*, confirming that passive acoustic monitoring supported by optimized signal processing can yield high-quality species-level data. These results are encouraging because amphibians are particularly sensitive to environmental change, and reliable monitoring of their vocal activity is essential for conservation and management.

### 4.1. Species Differences in Detection Performance

The higher accuracy for *D. japonicus* compared to *P. nigromaculatus* is likely attributable to differences in the acoustic structure of their calls. The advertisement call of *P. nigromaculatus* is characterized by relatively low-frequency, pulsed notes with clear harmonic energy, which in principle provides distinctive spectral features for separation from background noise. However, in practice, these calls occupy the same low-frequency bands that are frequently masked by anthropogenic and biotic noise (e.g., machinery, wind, other anurans), thereby reducing precision and recall. In contrast, *D. japonicus* produces shorter, higher-pitched calls that—although sometimes overlapping with insect choruses—were more consistently extracted by the NMF-based detector, resulting in higher overall performance.

Ecologically, this difference also mirrors broader life-history strategies. *P. nigromaculatus* often breeds in larger water bodies such as ponds and rice paddies, where low-frequency environmental noise and overlapping ranid frog choruses are common. Although its low-frequency calls are more easily masked by ambient noise from a human or technical perspective, this may not necessarily be disadvantageous for the frogs themselves. Low-frequency sounds can travel farther with less attenuation in open aquatic environments, allowing effective communication among conspecifics even over long distances, while potentially being less conspicuous to predators. Such trade-offs between communication range and acoustic conspicuousness could help explain why *P. nigromaculatus* maintains low-frequency vocalizations in these habitats. *D. japonicus*, by contrast, frequently calls from vegetated margins and forest edges, where acoustic clutter is present, but its tonal calls remain relatively distinct. The reduced detectability of *P. nigromaculatus* is therefore not only a technical limitation but also reflects ecological realities of habitat use and communication.

An important aspect of our findings is consistent with earlier observations that species constrained to low-frequency acoustic niches are especially vulnerable to masking [42,43]. In our study, although NMF-based source separation enhanced detection of *P. nigromaculatus* compared to what would be expected from traditional detectors, performance still lagged *D. japonicus*. This highlights both the promise and the limitations of automated approaches: signal processing can reduce bias against low-frequency callers, but ecological masking remains a fundamental constraint. Reliable detection across species therefore requires integrating technical solutions with an understanding of species-specific acoustic ecology.

### 4.2. Implications for Amphibian Bioacoustics

Amphibian vocalizations are closely tied to reproductive ecology. Accurate detection enables detailed assessment of breeding phenology, diel activity patterns, and relationships with abiotic drivers such as temperature and humidity. Our findings align with previous reports that calling activity in *D. japonicus* and *P. nigromaculatus* is strongly temperature dependent, often initiating shortly after dusk and increasing with ambient warmth [1,2]. Reliable classification across species therefore ensures that temperature–calling relationships can be modeled without bias introduced by systematic misclassification.

Our modeling analyses revealed clear interspecific contrasts in responses to weather drivers. While both species increased calling activity with rising temperature, *D. japonicus* exhibited much higher predicted call outputs under warm conditions, whereas *P. nigromaculatus* peaked at substantially lower levels. These differences likely reflect a combination of physiological and behavioral strategies, as well as ecological contexts such as male–male competition and habitat acoustics, which can influence total call output.

The contrasting temperature-calling responses of two species are broadly consistent with previous studies of frog calling ecology. Elevated evening temperatures have been shown to initiate and intensify calling in hylid frogs [1,2], consistent with the strong thermal dependence observed in *D. japonicus*. In contrast, *P. nigromaculatus* exhibits dual sensitivity to both temperature and moisture availability [44], paralleling our results, whereas *D. japonicus* shows a more unidirectional dependence on temperature [35]. These comparisons confirm that the divergent climatic sensitivities identified in our ZINB models are not artifacts of the dataset but reflect broader, repeatable species-level strategies. Although we hypothesized that lower-latitude sites would initiate calling earlier, we did not observe significant among-site differences in phenological timing. A likely explanation is that water was introduced to rice paddies at similar times across regions due to coordinated agricultural schedules, synchronizing breeding onset despite latitudinal variation.

Temperature emerged as a key predictor of calling activity, consistent with earlier studies showing that higher ambient temperatures accelerate calling onset and increase calling rate [45,46,47]. Rainfall and humidity, although not explicitly modeled here, are also known to trigger mass chorusing in hylid and ranid frogs. These results highlight the sensitivity of amphibian breeding phenology to short-term weather variation and emphasize the value of long-term passive acoustic monitoring for detecting climate-driven changes [3,4].

In summary, the combination of automated detection and ecological modeling provides both methodological and biological insights: it demonstrates that scalable monitoring tools can yield reliable species-level call data, and it reveals species-specific climatic sensitivities that have implications for predicting how amphibian breeding phenology may shift under future climate scenarios.

### 4.3. Broader Ecological and Conservation Significance

Amphibians are globally recognized as bioindicators due to their permeable skin, biphasic life histories, and reliance on both aquatic and terrestrial habitats. Declines in amphibian populations are often among the earliest warnings of ecosystem degradation [4]. Automated acoustic monitoring provides a cost-effective, noninvasive tool to track these populations at scales not possible with traditional survey methods. By showing that relatively simple signal processing approaches can achieve high detection accuracy, our study underscores that technological barriers to large-scale amphibian monitoring are diminishing.

The implications extend to landscape ecology. High-quality detection data allow quantification of how calling activity responds to environmental gradients such as land use, water quality, and climate variability. For instance, increasing temperatures under climate change may alter breeding phenology, leading to temporal mismatches between calling activity and resource availability. Reliable acoustic detection is thus not only a methodological advance but also a prerequisite for understanding how amphibians will respond to rapid global change.

### 4.4. Limitations and Future Research

Despite the promising results, several limitations must be considered. First, our study focused on two species in a specific geographic region, and generalization to other taxa or habitats requires further validation. Many amphibians produce calls with lower signal-to-noise ratios or more complex temporal patterns, which may necessitate alternative parameter tuning or hybrid approaches incorporating deep learning. Second, our analysis did not estimate abundance or density, as passive acoustic detection alone cannot readily distinguish numbers of individuals without spatial calibration. Integration with spatially explicit monitoring, such as microphone arrays or distance sampling, would be necessary to advance from presence/absence to population size inference. Finally, environmental noise remains a key challenge and developing adaptive algorithms that can account for dynamic soundscapes will be essential.

## 5. Conclusions

Using five years of passive acoustic monitoring with automated detection, we established a phenological baseline for two common frogs in Korean rice paddies. *Dryophytes japonicus* showed a short, synchronized seasonal peak centered in June, whereas *Pelophylax nigromaculatus* began earlier and maintained a broader, less synchronized calling period. Calling rate tended to be higher at southern sites, while onset timing differed only slightly among locations. These patterns indicate that agricultural wetlands support predictable, species-specific reproductive schedules that can be tracked reliably with automated methods.

Temperature emerged as the principal correlate of calling activity in both species, with additional weather variables contributing to species- and context-dependent ways. Model results suggest that humidity can modify temperature effects differently across species; for example, *P. nigromaculatus* tended to call less under combined warm and humid conditions, while *D. japonicus* maintained relatively high activity in warm weather with only modest attenuation at high humidity. Together, these findings provide a practical framework for long-term bioacoustic monitoring in agroecosystems and a reference point for detecting future phenological shifts. Further work that integrates hydrological management data, expands taxonomic and geographic coverage, and calibrates acoustic activity to abundance would strengthen inferences about population change and the mechanisms linking climate and amphibian reproduction.

## Figures and Tables

**Table 1:** Total number of valid recording days per year at each study site (Dangjin, Buan and Haenam) from 2018 to 2022.

Year	Dangjin	Buan	Haenam	Total
2018	45	88	89	222
2019	46	35	22	103
2020	177	104	158	439
2021	178	169	181	528
2022	171	161	174	506

**Table 2:** Confusion matrix for *D. japonicus* and *P. nigromaculatus*. The annotated subset consisted of 10% training and 90% testing recordings used for parameter optimization and validation, respectively.

		Predicted Positive	Predicted Negative
*D. japonicus*	Actual Positive	3042 (True Positive)	702 (False Negative)
	Actual Negative	702 (False Positive)	6354 (True Negative)
*P.* *nigromaculatus*	Actual Positive	935 (True Positive)	1404 (False Negative)
	Actual Negative	1404 (False Positive)	7057 (True Negative)

**Table 3:** Site-specific phenology thresholds (mean ± SD, Julian day) for *D. japonicus* and *P. nigromaculatus*. First detection values are calculated from 2020–2022 data only, due to missing observations in earlier years. Onset = first day at which 10% of cumulative seasonal calls were reached, Peak = 50%, and Termination = 90%.

Species	Site	First Detection	Onset	Peak	Termination
*D. japonicus*	Dangjin	83.7 ± 2.5	136.2 ± 1.5	167.6 ± 2.1	199.4 ± 4.6
	Buan	81.0 ± 10.0	134.8 ± 2.3	166.0 ± 2.5	196.8 ± 5.1
	Haenam	76.3 ± 5.7	134.0 ± 2.4	166.0 ± 2.7	198.6 ± 4.5
*P.* *nigromaculatus*	Dangjin	100.3 ± 7.5	131.6 ± 1.9	157.0 ± 1.0	180.4 ± 3.2
	Buan	105.3 ± 16.4	129.8 ± 1.9	156.2 ± 1.3	179.0 ± 5.1
	Haenam	92.7 ± 6.5	129.0 ± 2.4	155.6 ± 1.5	180.0 ± 4.0

## Data Availability

Weather variables were obtained from the RDA Open Data Portal (Observation service: https://weather.rda.go.kr/weather/observation.do; accessed on 1 March 2025). We used the soundscape-IR pipeline released by Lin (repository: schonkopf/soundscape_IR; see also meil-brcas-org/soundscape_IR for releases). Minor species-specific parameterization and small code adjustments were applied. The modified soundscape-IR code is available from the corresponding author upon reasonable request, and the original repository is cited in the References. Due to site sensitivity (wetland locations and species’ presence), the possibility of incidental human speech in field recordings, and file-size constraints, raw acoustic recordings are not publicly posted; they can be provided by the corresponding au-thor upon reasonable request and an appropriate data-use agreement. The R and Python scripts used in this study, together with a sample dataset, are available from the corresponding author upon reasonable request.
